# How Imitation Learning and Human Factors Can Be Combined in a Model Predictive Control Algorithm for Adaptive Motion Planning and Control

**DOI:** 10.3390/s21124012

**Published:** 2021-06-10

**Authors:** Milad Karimshoushtari, Carlo Novara, Fabio Tango

**Affiliations:** 1 Deparment of Electronics and Telecommunications, Politecnico di Torino, 10129 Torino, Italy; carlo.novara@polito.it; 2 Centro Ricerche Fiat, 10043 Torino, Italy; fabio.tango@crf.it

**Keywords:** trajectory planning, vehicle dynamics control, Model Predictive Control, learning, overtaking maneuver

## Abstract

Interest in autonomous vehicles (AVs) has significantly increased in recent years, but despite the huge research efforts carried out in the field of intelligent transportation systems (ITSs), several technological challenges must still be addressed before AVs can be extensively deployed in any environment. In this context, one of the key technological enablers is represented by the motion-planning and control system, with the aim of guaranteeing the occupants comfort and safety. In this paper, a trajectory-planning and control algorithm is developed based on a Model Predictive Control (MPC) approach that is able to work in different road scenarios (such as urban areas and motorways). This MPC is designed considering imitation-learning from a specific dataset (from real-world overtaking maneuver data), with the aim of getting human-like behavior. The algorithm is used to generate optimal trajectories and control the vehicle dynamics. Simulations and Hardware-In-the-Loop tests are carried out to demonstrate the effectiveness and computation efficiency of the proposed approach.

## 1. Introduction

In recent years, as a consequence of the fact that safety aspects have gained huge importance in society and in the transportation industry, the advent of automated driving systems (ADSs) marks one of the biggest events in transportation research. In fact, traffic volumes are constantly increasing (at least they had been before the COVID-19 pandemic) and the number of vehicle accidents has also equally increased, with large effects on people’s safety and quality of life, as well as on social financial expenses. As claimed in [1], in 2016, for example, the World Health Organization estimated that the number of deaths related to road accidents was over 3400 per day, with associated costs having an impact of nearly 3% of the world’s Gross Domestic Product. In this picture, it is assumed that autonomous cars can reduce the quantity of road accidents and injuries, improving traffic operations and making them easier. In addition, these autonomous vehicles (AVs) will enable aged or disabled people to go anywhere, independently, at any time.

Despite the clear advantages that intelligent transportation systems (ITSs) can offer and the huge research efforts carried out in this field, several technological challenges must still be addressed, because AVs require deep information about the surroundings and the identification of route/trajectory planning. In particular, AVs require motion-planning methods to generalize unpredictable situations in a timely manner, presenting smooth behavior in order to guarantee comfort, safety and efficiency to the vehicle’s occupants.

Different approaches to motion/trajectory planning can be found in the literature, including graph-based algorithms (A*, D*, Theta*, etc.), Artificial Potential Field (APF) techniques, probabilistic methods (Probabilistic Road Maps (PRM), several variants of rapid-exploring random trees (RRT), etc.), Voronoi Diagram techniques, and so on. A* and its variants are based on weighted graphs and they aim to find an optimal path from the starting node to the target note, trying to minimize suitable costs (e.g., the distance traveled) [2]. D* is a dynamic version of A*, where the graph weights may change in time [3]. Theta* is a variant of A* that propagates information along the graph edges without constraining the paths to the edges [4]. APF techniques are based on the design of a suitable potential field so that the vehicle, subjected to the force obtained from this potential, reaches its goal, avoiding possible obstacles [5]. PRM methods proceed in two phases: a learning phase and a query phase [6]. In the learning phase, a probabilistic road map is first constructed. The roadmap is then stored as a graph, where the vehicle configurations are the nodes and the possible paths are the edges. In the query phase, the path is obtained by a suitable search algorithm (e.g., A*). RRT and its variants are based on the construction of a random space-filling tree [7]. The tree is generated incrementally in such a way as to grow towards unexplored regions. Voronoi Diagram techniques allow the generation of a road map with a maximum space from the obstacles, and the corresponding graph is fully connected [8]. Given this map, the path is planned by a suitable search algorithm (e.g., A*). All these methods are typically used for global path planning, that is, planning of a suitable path between two points on a given map. This planning is carried out at the beginning of the trip and possibly updated in the case of changes of the road scenario. Global path planning often requires subsequent local trajectory planning, which is carried out on-line in order to provide smooth trajectories which can better adapt to the road scenario near the vehicle. In any case, both global and local plannings provide a geometric path, which is generally inconsistent with the vehicle dynamics. As a consequence, it may be hard for the vehicle to track the planned path.

Once a suitable trajectory has been planned, a control action is needed to make the vehicle track such a trajectory. This task is accomplished by a feedback control system which, on the basis of the current vehicle state and the planned trajectory, provides the throttle and steering commands, allowing trajectory tracking. Several control approaches can be found in the literature, including the Proportional Integral Derivative (PID), Stanley controller, Eigenvalue Placement, Linear Quadratic Regulator (LQR) and its version with the integral action (LQI), Gain Scheduling, Sliding Mode Control, Adaptive Control, and Model Predictive Control (MPC) (see, e.g., [9,10,11,12,13,14,15,16] and the references therein). It can be noted that in the majority of these approaches, trajectory planning and vehicle control are carried out separately, resulting in possibly non-optimal vehicle motion.

In this paper, a MPC approach is adopted. MPC is a general methodology for control and trajectory optimization of complex linear and nonlinear dynamic systems. This methodology is based on two key operations: (1) prediction of the behavior of the system of interest over a finite time interval; and (2) optimization of the system trajectory, based on its predicted behavior, and calculation of the optimal trajectory and control law. The main features/advantages of MPC are the following:

- MPC jointly performs (local) trajectory planning and control. As mentioned above, the optimization process provides a suitable vehicle trajectory together with the control command, making the vehicle track the trajectory.
- The planned trajectories are:
  – Optimal (over a finite time interval). In fact, they are obtained as solutions of a suitable optimization problem.
  – Consistent with the vehicle dynamics. A constraint is directly imposed in the optimization problem, forcing the trajectories to satisfy the vehicle dynamics/ kinematics equations.
- Trajectory planning is performed on-line. This allows the ego vehicle to adapt in real time to the road scenario and to promptly react when unexpected events occur.
- MPC can systematically deal with constraints. Besides the dynamics/kinematics constraint, other constraints can be inserted in the optimization problem, which can account for command saturations, obstacles which may affect the trajectory, boundaries in the trajectory domain, and so forth.
- MPC can efficiently manage the trade-off between performance and energy consumption. Indeed, trajectory planning is attained by minimization of an objective function consisting of two terms describing the maneuver precision, and one term quantifying the command effort (that is related to energy consumption). These terms are characterized by suitable weight matrices, which can be designed to systematically manage the aforementioned trade-off.

To the best of our knowledge, MPC is the only method characterized by all these features together. As discussed above, most state-of-the-art approaches cannot perform trajectory planning and control jointly. These two tasks are typically carried out separately, and this may result in non-optimal vehicle motion. Moreover, the planned trajectory may not be fully consistent with the vehicle dynamics/kinematics, and this may lead to unexpected behaviours of the vehicle and/or ineffective obstacle avoidance.

In order to properly cope with the nonlinear behavior of the vehicle dynamics, a Nonlinear MPC (NMPC) algorithm is developed in this paper. A key innovative feature of the algorithm is that its parameters are learned from experimental data collected in real scenarios (i.e., lane-change and overtaking maneuvers in motorway conditions). The motivation for this choice is to make the planning behavior more comfortable and more human-like, thus facilitating the acceptability and usability of AVs.

Another important feature is the numerical efficiency of the NMPC algorithm. Indeed, a general drawback common to most MPC approaches is the high computational cost. Nevertheless, the algorithm developed here is characterized by a relatively low computational cost, allowing its real-time implementation on the embedded processors that are used in automotive applications.

Thanks to this feature, another innovation of the proposed NMPC algorithm consists of the applicative perspective: the algorithm will be implemented and used in real-time on a real prototype car, within the European project PRYSTINE [17] (in the use-cases of this project, the required sampling time is 100 ms or lower).

## 2. Vehicle Prototype Description and Model

In this section, the use-case is presented and, moreover, Section 2.1 and Section 2.2 provide a description of the test-vehicle and of the related experiments. In addition, Section 2.4 gives an overview of the model that is used.

### 2.1. Use-Cases Definition and Description

**Figure 1:**
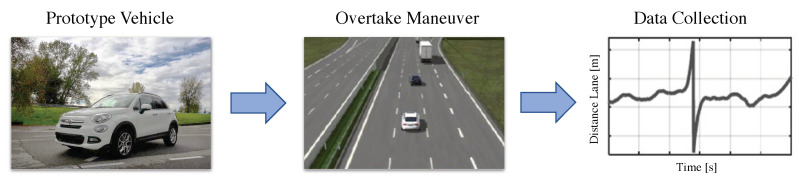
Type of tests for real-data collection.

In order to collect the dataset used for the development and training of the MPC algorithm, we defined the following use-case: the host-vehicle is approaching a slower object (i.e., another vehicle) ahead in a motorway scenario, and therefore an overtaking maneuver is needed. The situation is sketched in Figure 1.

### 2.2. Prototype Vehicle

**Figure 2:**
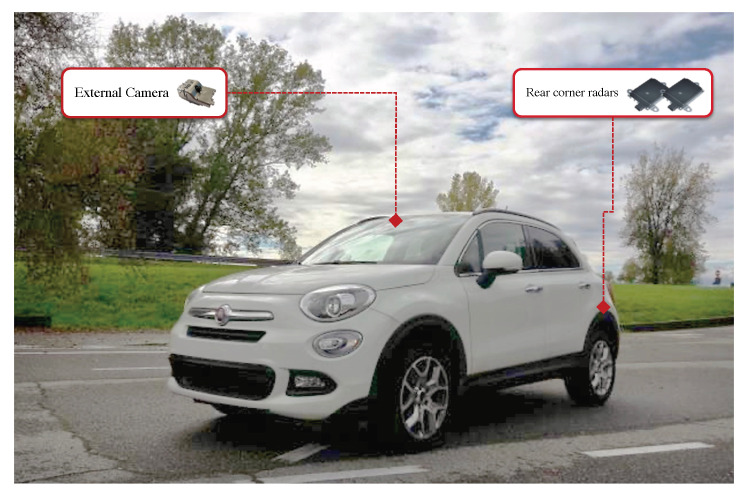
Prototype vehicle with installed sensors.

In order to prepare the dataset for algorithm training, a data-logger was prepared and a dedicated experimental phase was executed by using a specifically equipped prototype vehicle shown in Figure 2.

The prototype vehicle is a “Fiat 500x 1.6 Mjet” (120 cv–LOUNGE version), equipped with the following components:

- External camera, with a Field of View (FoV) of 52${}^{\circ }$, to detect obstacles and lanes on the road.
- External rear-corner radars, with a FoV of 15$0{}^{\circ }$ (±75${}^{\circ }$) and maximum distance of 90 m, to detect objects coming from the rear, in the adjacent lane.
- The dSpace MicroAutoBox II is used to manage the CAN board, in particular to control the data synchronization and acquisition; the control panel is used to activate the logging system and the start-up of sensors (cameras, above all).

### 2.3. Experimental Phase

The procedure for the experiments is described as follows. Ten users participated in the experiments. When asked by the experimenter, they were told to execute a specific maneuver of overtaking. The full maneuver was labelled in different sequences:

1. Approaching the (slower) vehicle ahead (car following/approaching);
2. Left lane change (LC);
3. Passing the vehicle aside, travelling on the right lane (this can also be multiple passing, if more than one vehicle is present);
4. Right LC;
5. Lane-keeping (LK) or free-riding—means the end of the overtaking maneuver.

The following data were collected during each experiment:

- Vehicle dynamics:
  – Speed;
  – Steering wheel;
  – Yaw rate;
  – Acceleration;
  – Brake and accelerator pedal positions.
- Road information:
  – Number and type of lanes;
  – Road curvature;
  – Variation of road curvature (when present);
  – Position of the ego-vehicle in the lane;
  – Heading angle.
- Environmental information (of obstacles):
  – (Relative) speed;
  – Distance;
  – Angular position.

**Figure 3:**
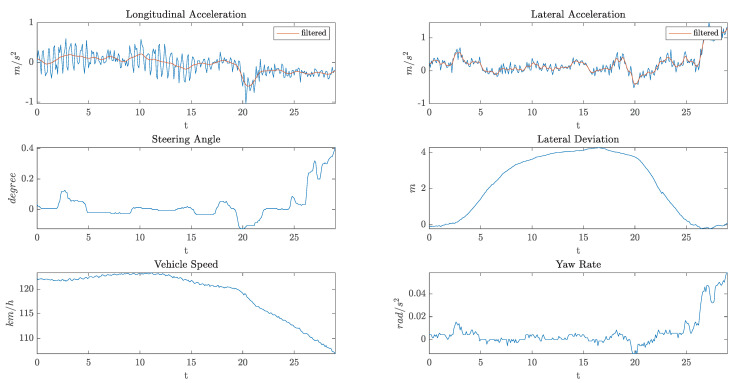
An example of the data collected by the on-board sensors.

Figure 3 shows an example of the data collected by the on-board sensors during an overtaking maneuver.

**Figure 4:**
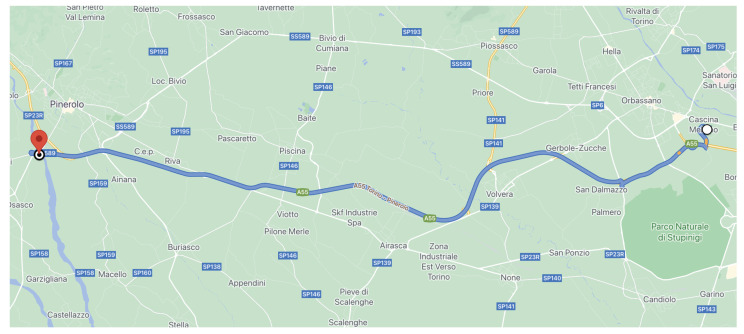
Test site around CRF location for the data collection on overtaking maneuver.

Figure 4 describes the test site considered in these experiments:

The test site starts from the CRF location in Orbassano, arrives at Pinerolo town (following A55 motorway), and then comes back. It included two scenarios: motorway and extra-urban roads (both with two lanes for each carriage). The total length was 58.6 km. Then, the related datasets were post-processed (to remove invalid data, correct the labels manually written down, and so on) to be used for parameter-tuning of the MPC algorithm.

### 2.4. Single-Track Model

In this section, a standard model of the lateral and longitudinal dynamics of a vehicle is presented, called the dynamic single-track model [11]. Although simple, this model captures the main aspects of the vehicle dynamics and, for this reason, it is suitable for the design and preliminary tests of vehicle control systems. It can be mentioned that the single-track model is often called a "bicycle model" in the literature. In the following, the model will be named the Dynamic Single-Track with Pacejka’s formula model (DSTP) for short. The model will be used for the design of the MPC trajectory-planning and control algorithm.

**Figure 5:**
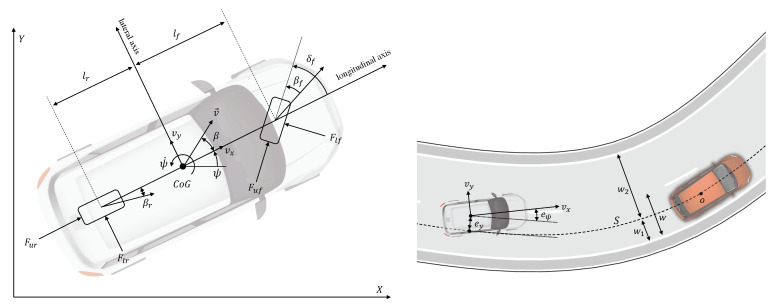
(**left**) Single-track model, (**right**) path-tracking errors.

The DSTP model variables and parameters are the following (see Figure 5):

- Vehicle variables:
  X,Y: coordinates of the vehicle’s center of gravity (CoG) in an inertial reference frame;
  ψ: yaw angle;
  $\dot{\psi }$: yaw rate;
  $\vec{v}\equiv V$: velocity vector in the inertial frame;
  $v_{x}$: longitudinal speed = $\vec{v}$ component along the longitudinal axis;
  $v_{y}$: lateral speed = $\vec{v}$ component along the transverse axis;
  $a_{x}$: longitudinal acceleration;
  $\delta_{f}$: front wheel steering angle;
  β: vehicle slip angle = angle between the vehicle longitudinal axis and velocity;
  $\beta_{f},\beta_{r}$: tire slip angles = angles between the tires’ longitudinal axis and velocity.
- Vehicle parameters:
  $m,I_{z}$: mass and yaw polar inertia;
  $l_{f}$: distance CoG - front wheel center;
  $l_{r}$: distance CoG - rear wheel center;
  $l_{w}$: vehicle width;
  $c_{f},c_{r}$: front/rear cornering stiffnesses;
  $\eta_{f},\eta_{r}$: front/rear vertical load factors.

The state equations of the DSTP model are:

(1) $$\begin{array}{cc} \; & \dot{X}=v_{x}\cos\psi -v_{y}\sin\psi \\ \; & \dot{Y}=v_{x}\sin\psi +v_{y}\cos\psi \\ \; & {\dot{v}}_{x}=v_{y}\dot{\psi }+a_{x}\\ \; & {\dot{v}}_{y}=-v_{x}\dot{\psi }+\frac{2}{m}(F_{yf}+F_{yr})\\ \; & \ddot{\psi }=\frac{2}{I_{z}}(l_{f}F_{yf}-l_{r}F_{yr}). \end{array}$$

$F_{xf}$, $F_{yf}$, $F_{xr}$, and $F_{yr}$ are longitudinal and lateral forces between the wheels and the vehicle.

(2) $$\begin{array}{cc} \; & a_{x}=\frac{2}{m}\left(F_{xf}+F_{xr}\right)\\ \; & F_{xf}=F_{uf}\cos\left(\delta_{f}\right)-F_{lf}\sin\left(\delta_{f}\right)\\ \; & F_{yf}=F_{uf}\sin\left(\delta_{f}\right)+F_{lf}\cos\left(\delta_{f}\right)\\ \; & F_{xr}=F_{ur}\\ \; & F_{yr}=F_{lr}, \end{array}$$

where $F_{lf}$ and $F_{lr}$ are the lateral forces exchanged between the tire and road. The following tire force model is considered (see [11]):

$$\begin{array}{ccc} F_{lf}=-f_{P}\left(\beta_{f}\right) & & F_{lr}=-f_{P}\left(\beta_{r}\right)\;\\ \beta_{f}=\mathrm{atan}\left(\frac{v_{y}+l_{f}\dot{\psi }}{v_{x}}\right)-\delta_{f} & & \beta_{r}=\mathrm{atan}\left(\frac{v_{y}-l_{r}\dot{\psi }}{v_{x}}\right). \end{array}$$

**Figure 6:**
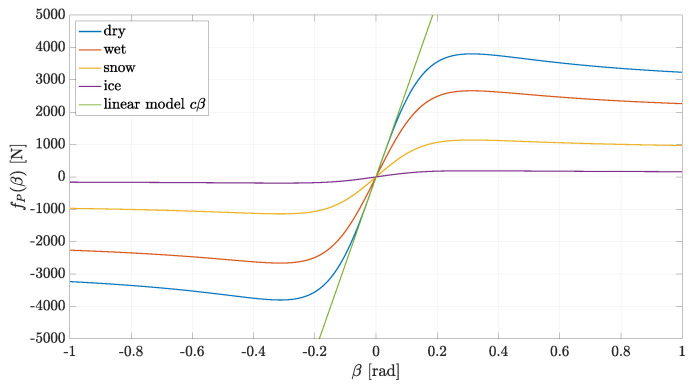
Pacejka’s tire model.

$f_{P}\left(\beta \right)$ is given by Pacejka’s magic formula:

$$\begin{array}{cc} f_{P}\left(\beta \right) & ≐p_{1}\sin\left(p_{2}\;\mathrm{atan}\left(p_{3}\beta -p_{4}\left(p_{3}\beta -\mathrm{atan}\left(p_{3}\beta \right)\right)\right)\right) \end{array},$$

where $p_{1}$ is the peak value, $p_{2}$ is the shape factor, $p_{3}$ is the stiffness factor and $p_{4}$ is the curvature factor. Pacejka’s tire model is illustrated in Figure 6 for different road conditions.

The model (1) will be used in Section 3.2 to design a Trajectory Planning and Control (TPC) algorithm for an ego vehicle. To this aim, it is also convenient to introduce the following path-tracking errors (see Figure 5):

- $e_{y}$ (lateral error): lateral deviation of the vehicle CoG from the reference path *S*.
- $e_{\psi }$ (orientation error): angular deviation between the vehicle orientation and the direction of the reference path *S*.

The time evolution of these errors is described by the following equations (see [11]):

(3) $$\begin{array}{cc} \; & {\dot{e}}_{y}=v_{y}+v_{x}e_{\psi }\\ \; & {\dot{e}}_{\psi }=\dot{\psi }-v_{x}\rho_{S}, \end{array}$$

where $\rho_{S}$ is the local curvature of the reference path *S*. Equations (1) and (3) constitute the basic model for the TPC algorithm that will be proposed in Section 3.2. The state of this model is $x=(X,Y,v_{x},v_{y},\psi ,\dot{\psi },e_{y},e_{\psi })$, whereas the input is $u=(a_{x},\delta_{f})$.

## 3. Methods

In this section, a general formulation of the Nonlinear Model Predictive Control (NMPC) approach is presented. This approach will be adopted in Section 3.2 to design and implement a Trajectory Planning and Control (TPC) algorithm.

### 3.1. NMPC General Formulation

NMPC is a general and flexible approach to control the complex nonlinear systems [18,19,20]. NMPC provides optimal solutions (over a finite time-interval), can deal with input/state/output constraints, and can systematically manage the trade-off between performance and command activity. Successful applications of NMPC can be found in many areas, such as automotive engineering, aerospace engineering, chemical process management, robotics, biomedicine, and so forth. Here, a concise but self-contained formulation of NMPC is provided.

Consider a Multiple-Input-Multiple-Output (MIMO) nonlinear system described by the following state equations:

(4) $$\begin{array}{cc} \dot{x}= & f(x,u)\\ y= & h(x,u), \end{array}$$

where $x\in R^{n_{x}}$ is the state, $u\in R^{n_{u}}$ is the command input and $y\in R^{n_{y}}$ is the output; $f:R^{n_{x}+n_{u}}\to R^{n_{x}}$ and $g:R^{n_{x}+n_{u}}\to R^{n_{y}}$ are two functions characterizing the system dynamics and output variables, respectively. Assume that the state is measured in real-time, with a sampling time $T_{s}$, according to

$$x\left(t_{k}\right),\;t_{k}=T_{s}k,\;k=0,1.\ldots$$

If the state is not measured, an observer has to be employed, or a model of (4) in input–output form.

Suppose that the system output y(t) is required to track a desired reference signal r(t). The state, output and input variables may be subject to constraints, and it may be of interest to have a suitable trade-off between performance and command effort.

NMPC is a suitable approach to tackle such a control problem and it is based on two key operations: prediction and optimization. At each time $t=t_{k}$, the system state and output are predicted over the time interval $\left[t,t+T_{p}\right]$, where $T_{p}\geq T_{s}$ is called the *prediction horizon*. The prediction is obtained by integration of the model Equation (4). For any $\tau \in [t,t+T_{p}]$, the predicted output $\hat{y}\left(\tau \right)$ is a function of the “initial” state x(t) and the input signal:

$$\hat{y}\left(\tau \right)\equiv \hat{y}\left(\tau ,x(t),u(t:\tau )\right),$$

where u(t:τ) denotes the input signal in the interval [t,τ]. The basic idea of NMPC (and of the most predictive approaches) is to look for an input signal $u^{*}\left(t:\tau \right)$ at each time $t=t_{k}$, such that the prediction $\hat{y}\left(\tau ,x\left(t\right),u^{*}\left(t:\tau \right)\right)$ has the desired behavior in the time interval $\left[t,t+T_{p}\right]$. The concept of desired behavior is formalized by defining the *objective function*

(5) $$J\left(u(t:t+T_{p})\right)≐\int_{t}^{t+T_{p}}\left({∥{\tilde{y}}_{p}\left(\tau \right)∥}_{Q}^{2}+{∥u(\tau )∥}_{R}^{2}\right)d\tau +{∥{\tilde{y}}_{p}\left(t+T_{p}\right)∥}_{P}^{2},$$

where ${\tilde{y}}_{p}\left(\tau \right)≐r\left(\tau \right)-\hat{y}\left(\tau \right)$ is the predicted tracking error, $r\left(\tau \right)\in R^{n_{y}}$ is the reference to track, and ${∥\;\cdot \;∥}_{*}$ is a weighted vector norm. For a generic vector *w* and a positive definite weight matrix *Q*, this norm is defined as ${∥w∥}_{Q}^{2}≐w^{⊤}Qw.$ In most cases, diagonal weight matrices are used, since the non-diagonal terms are generally difficult to manage/interpret and their utilization usually does not yield any relevant advantage.

The input signal $u^{*}\left(t:t+T_{p}\right)$ was chosen as one minimizing the objective function $J\left(u(t:t+T_{p})\right)$. In particular, at each time $t=t_{k}$, for $\tau \in [t,t+T_{p}]$, the following optimization problem is solved:

(6) $$\begin{array}{c} u^{*}\left(t:t+T_{p}\right)=\arg\min_{u(\;\cdot \;)}J\left(u(t:t+T_{p})\right)\\ \mathrm{subject}\mathrm{to}:\;\\ \;\dot{\hat{x}}\left(\tau \right)=f\left(\hat{x}\left(\tau \right),u\left(\tau \right)\right),\;\hat{x}\left(t\right)=x\left(t\right)\;\\ \;\hat{y}\left(\tau \right)=h\left(\hat{x}\left(\tau \right),u\left(\tau \right)\right)\;\\ \;\hat{x}\left(\tau \right)\in X_{c},\;\hat{y}\left(\tau \right)\in Y_{c},\;u\left(\tau \right)\in U_{c}, \end{array}$$

where $0\leq T_{s}\leq T_{p}$. The fist two constraints in this problem ensure that the predicted state and output are consistent with the system Equations (4). The sets $X_{c}$ and $Y_{c}$ account for other constraints that may hold for the predicted state/output (e.g., obstacles, barriers). The set $U_{c}$ accounts for input constraints (e.g., input saturation).

Note that the optimization problem (6) is generally non-convex. Moreover, the decision variable u(·) is a signal, and optimizing a function with respect to a signal is generally a difficult task. To overcome this problem, a suitable parametrization of the input signal *u* is taken. In particular, the prediction interval $\left[t,t+T_{p}\right]$ is divided into sub-intervals $\left[t+\tau_{i},t+\tau_{i+1}\right]\subset \left[t,t+T_{p}\right]$, $i\in \{1,2,\ldots ,n_{I}+1\}$, where the $\tau_{i}$s are called the nodes. Then, *u* is assumed constant on each sub-interval, so that the optimization problem reduces to a finite-dimension problem, which is solved using an efficient numerical optimization algorithm.

The NMPC feedback command is obtained by solving the optimization problem (6) at each sampling time $t=t_{k}$, according to a so-called receding horizon strategy:

- At time $t=t_{k}$:
  – Compute $u^{*}\left(t:t+T_{p}\right)$ by solving (6);
  – Apply to the system only the first input value: $u\left(\tau \right)=u^{*}\left(t_{k}\right)$ and keep it constant for $\forall \tau \in [t_{k},t_{k+1}]$;
- Repeat the two steps above for $t=t_{k+1},t_{k+2}.\ldots$

Such a receding horizon strategy is important in order to have a feedback control action, which may be crucial in order to stabilize unstable systems, attenuate external disturbances and properly react if sudden changes occur in the scenario where the system of interest is operating.

**Remark** **1.** An interesting feature of NMPC (and of its linear version) is its capability to jointly perform (local) trajectory planning and control. Indeed, the predicted state signal $\hat{x}\left(t:t+T_{p}\right)$ obtained by solving problem (6) is an optimal trajectory (over a finite time interval). The corresponding control input $u^{*}\left(t_{k}\right)$ is the command making the system track this optimal trajectory. Note that the optimal trajectory and command are computed in real time and updated at each sampling time according to the receding horizon strategy, allowing the vehicle to promptly adapt to possible road scenario variations.

### 3.2. TPC Design and Implementation

In this section, a trajectory planning and control (TPC) algorithm is designed, based on the NMPC approach described in Section 3.1 and then implemented on an embedded processor.

#### 3.2.1. TPC Design

**Figure 7:**
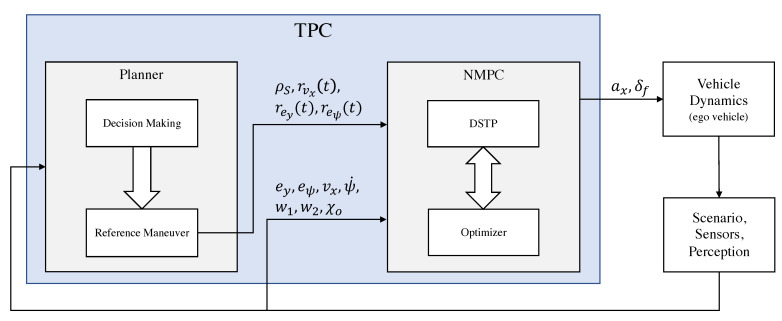
Block diagram of the TPC algorithm.

Consider the block diagram in Figure 7. We can distinguish the following blocks:

- *Ego vehicle*. Autonomous vehicle whose trajectory must be planned and controlled.
- *Scenario, Perception*. Provides road and obstacle information.
- *TPC*. Trajectory planning and control algorithm which includes the NMPC controller and a planner.

The main variables of the block diagram in Figure 7 are as follows:

- *S*: reference path
- $\rho_{S}$: curvature of the reference path
- $r_{v_{x}}$: reference speed
- $r_{e_{y}}$: reference lateral deviation
- $r_{e_{\psi }}$: reference heading angle deviation
- $u=(a_{x},\delta_{f})$: ego vehicle command input

The planner includes a decision-making block that is a state machine based on the Markovian Decision Process (MDP) which decides the maneuvers that the autonomous vehicle should execute depending on its current driving scenario. The set of maneuvers which are planned should take the rules of the road into account and the interactions with all static and dynamic objects in the environment. The set of decisions made must ensure vehicle safety and efficient motion through the environment. Although the definition of this block is out of the scope of this paper, in Section 3.3 a subset of the necessary conditions to execute an overtaking maneuver is presented. Based on the output of the decision-making block, we then calculate a path and velocity reference depending on the type of the maneuver (for the overtaking maneuver, see Section 3.3). Then, the computed references are given to the NMPC controller.

**Remark** **2.** *The reference path is a geometric curve indicating to the ego vehicle where to move in a two-dimensional domain. It is important to note that, in general, this kind of path is not consistent with the vehicle dynamics. On the contrary, the trajectory planned by the NMPC algorithm satisfies the vehicle dynamics equations. We can say that the NMPC provides a trajectory close to the desired path, and this trajectory is consistent with the vehicle dynamics.*

The NMPC is designed according to Section 3.1 with a slight modification of the objective function (5); instead of the second term u(τ), its derivative is used since we are interested in minimizing the amplitude of the jerk and steering speed instead of the acceleration and steering angle. The details of the NMPC design are as follows:

- Prediction model: DSTP Equations (1) and (3).
   state: $x=(X,Y,v_{x},v_{y},\psi ,\dot{\psi },e_{y},e_{\psi })$,
   output: $y=(v_{x},e_{y},e_{\psi })$,
   command input: $u=(a_{x},\delta_{f})$.
- Input constraints:
   $\delta_{f}\in \left[\frac{\pi }{6},\frac{\pi }{6}\right]$, $a_{x}\in \left[-5,3\right]$
- State/output constraints:
   road constraint: $-\left(w_{1}-l_{w}/2\right)<e_{y}<\left(w_{2}-l_{w}/2\right)$
   obstacle collision constraints: $\chi_{e}\notin \chi_{o}$
   where $\chi_{e}$ is the set of ego vehicle body geometry positions and $\chi_{o}$ is the set of all obstacle/vehicle body geometry position predictions.
- Sampling time: $T_{s}=0.1$
- Prediction horizon: $T_{p}=1$
- Input nodes: $\tau_{i}=0.5$
- Weight matrices: Q=diag(1,10,10), P=diag(0,0,0), R=diag(1,0.1).

#### 3.2.2. TPC Implementation

**Figure 8:**
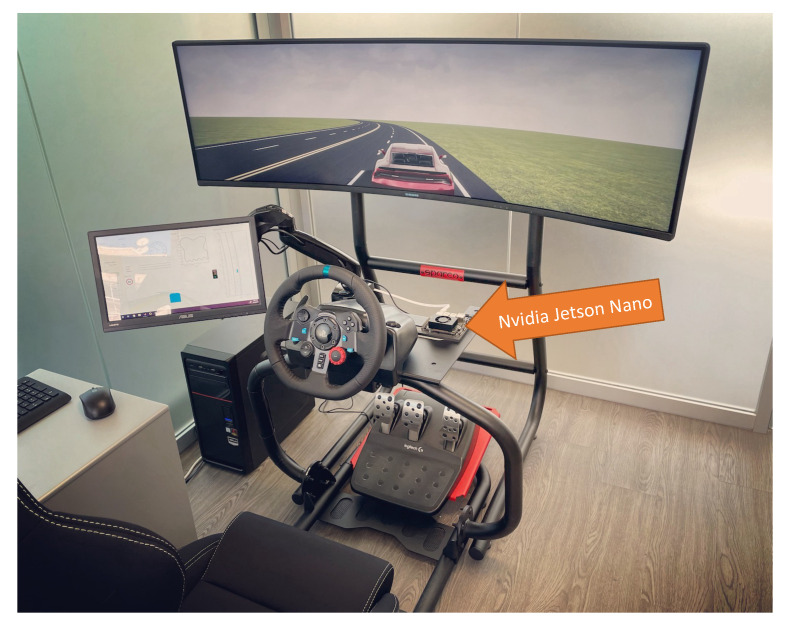
Hardware-in-the-loop (HIL) simulation.

The TPC algorithm has been implemented and tested in Matlab/Simulink, and hardware-in-the-loop tests were also performed using the Nvidia Jetson Nano board (see Figure 8).

In order to implement the algorithm on hardware, the algorithm was converted from a Matlab/Simulink code to C++ code by means of the Matlab/Simulink automatic code-generation tool. Then, the C++ code was deployed and built on the Jetson Nano board. Hardware-in-the-loop tests were carried out to validate the algorithm, where the vehicle was simulated in real-time on a PC, using the Matlab Automated Driving toolbox and Simulink Desktop Real-Time, while the TPC algorithm was running on the Jetson board.

The tests showed that the algorithm is computationally efficient to make its implementation possible on an embedded processor and executed in real-time. More specifically, the Jetson Nano board has a Quad-core ARM A57 processor with a clock speed of 1.43 GHz, and the time required by a single core of the processor to compute the optimal trajectory and the related command inputs is 25±5 ms, allowing adequate sampling time for the algorithm (typically, sampling times of 50 to 100 ms are adequate for effective vehicle dynamics control).

It is worth mentioning that the computation time of the algorithm on a PC with a core i7-7700 3.6 GHz processor was 20±6 ms, which is not much different from the one obtained with the embedded processor. Indeed, even though the PCs processor is much more powerful, the code running on the embedded processor is optimized and compiled into machine language unlike the PC, where the code runs in Matlab without compilation.

For comparison, a state-of-the-art approach for on-line trajectory planning and control has been considered. The approach is based on A* (trajectory planning), PID (longitudinal control) and Stanley (lateral control). On the same PC described above, using the A* algorithm of Matlab, the approach took 260±150 ms for computing the optimal trajectory and the related command inputs.

### 3.3. Overtaking Maneuver

Overtaking is one of the most frequently used and challenging maneuvers for AVs. Although there are several papers regarding the design of an overtaking trajectory [21,22,23], as discussed in the introduction, the majority of these approaches, such as trajectory planning and vehicle control, are carried out separately, resulting in possibly non-optimal vehicle motion. In this section, we parameterize the overtaking maneuver, and in the next section the parameters are learned from real-world experimental data in order to imitate human behaviour.

**Figure 9:**
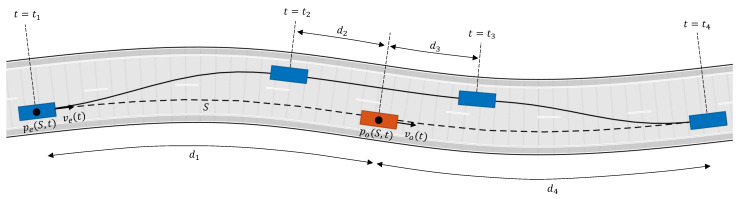
Overtaking maneuver, Phase 1: $\forall t\in [t_{1},t_{2})$, Phase 2: $\forall t\in [t_{2},t_{3}]$, Phase 3: $\forall t\in (t_{3},t_{4}]$.

The overtaking maneuver consists of three phases. Phase 1 is diversion from the lane, Phase 2 is driving straight in the adjacent lane, and Phase 3 is returning to the lane (see Figure 9). $p_{e}\left(S,t\right)$ and $p_{o}\left(S,t\right)$ are the positions of the ego vehicle and the overtaken vehicle projected along path *S* (path *S* is the center of the right lane in Figure 9, and δp(t) is the path length between $p_{e}\left(S,t\right)$ and $p_{o}\left(S,t\right)$. In order to determine the beginning and ending of each phase, we can parameterize the overtaking maneuver as follows:

- Phase 1 starts if $\delta p\left(t\right)<d_{1}$
- Phase 2 starts if $\delta p\left(t\right)<d_{2}$
- Phase 3 starts if $\delta p\left(t\right)>d_{3}$
- Phase 3 ends if $\delta p\left(t\right)>d_{4}$

The distances $d_{i},\;i=1,2,3,4$ are not constant. A reasonable assumption is then to consider them as functions of ego vehicle speed ($v_{e}$):

(7) $$d_{i}=k_{i}v_{e}\left(t_{i}\right)\;\mathrm{for}\;i=1,2,3,4.$$

The speed of the ego vehicle in Phase 2 must be greater than the speed of the overtaken vehicle $v_{o}\left(t\right)$. Therefore,

(8) $$\begin{array}{cc} \; & \forall t\in [t_{2},t_{3}]\\ \; & v_{e}\left(t\right)=v_{o}\left(t\right)+\delta v\;\mathrm{if}\;v_{e}\left(t\right)<v_{o}\left(t\right)+\delta v\\ \; & v_{e}\left(t\right)=v_{e}\left(t_{1}\right)\;\mathrm{if}\;v_{e}\left(t\right)\geq v_{o}\left(t\right)+\delta v. \end{array}$$

Finally, the vehicle must return to its original speed at the end of Phase 3:

(9) $$v_{e}\left(t_{4}\right)=v_{e}\left(t_{1}\right).$$

We can compute the reference acceleration as follows:

(10) $$\begin{array}{cc} \; & a\left(t\right)=\min\left(\bar{a},\frac{{\left(v_{e}\left(t_{2}\right)-v_{o}\left(t\right)\right)}^{2}-{\left(v_{e}\left(t\right)-v_{o}\left(t\right)\right)}^{2}}{2(\delta p\left(t\right)-d_{2})}\right)\;\forall t\in \left[t_{1},t_{2}\right)\\ \; & a\left(t\right)=0\;\forall t\in \left[t_{2},t_{3}\right]\\ \; & a\left(t\right)=\max\left(\underline{a},\frac{{\left(v_{e}\left(t_{4}\right)-v_{o}\left(t\right)\right)}^{2}-{\left(v_{e}\left(t\right)-v_{o}\left(t\right)\right)}^{2}}{2(d_{4}-\delta p\left(t\right))}\right)\;\forall t\in \left(t_{3},t_{4}\right], \end{array}$$

where $\bar{a}$, $\underline{a}$ are the maximum and minimum acceleration during the overtaking maneuver. Let’s assume the parameters $k_{1}$, $k_{2}$, $k_{3}$, $k_{4}$, δv, $\bar{a}$, and $\underline{a}$ are known; in the next section, we use real overtaking data to learn these parameters. Then, we compute the time required to finish each phase, which is denoted by T(t):

(11) $$\begin{array}{cc} \; & \delta p\left(t\right)-d_{2}=\frac{1}{2}a\left(t\right)T{\left(t\right)}^{2}+\left(v_{e}\left(t\right)-v_{o}\left(t\right)\right)T\left(t\right)\;\forall t\in \left[t_{1},t_{2}\right)\\ \; & d_{3}+\delta p\left(t\right)=\left(v_{e}\left(t\right)-v_{o}\left(t\right)\right)T\left(t\right)\;\forall t\in \left[t_{2},t_{3}\right]\\ \; & d_{4}-\delta p\left(t\right)=\frac{1}{2}a\left(t\right)T{\left(t\right)}^{2}+\left(v_{e}\left(t\right)-v_{o}\left(t\right)\right)T\left(t\right)\;\forall t\in \left(t_{3},t_{4}\right]. \end{array}$$

Finally, the references for the NMPC controller are computed as follows: the reference speed ($r_{v_{x}}$) is computed according to Equations (8)–(10), and the reference lateral deviation and heading angle ($r_{e_{y}},r_{e_{\psi }}$) are computed as follows:

(12) $$\begin{array}{c} \begin{array}{ccc} \; & r_{e_{y}}\left(t\right)=e_{y}\left(t\right)+\left(L-e_{y}\left(t\right)\right)\left(10{\left(\frac{t}{T(t)}\right)}^{3}-15{\left(\frac{t}{T(t)}\right)}^{4}+6{\left(\frac{t}{T(t)}\right)}^{5}\right) & \forall t\in \{\left[t_{1},t_{2}\right)\cup \left(t_{3},t_{4}\right]\}\\ \; & r_{e_{y}}\left(t\right)=w & \forall t\in [t_{2},t_{3}]\\ \; & r_{e_{\psi }}\left(t\right)=\arctan\left(\frac{r_{e_{y}}\left(t+T_{s}\right)-r_{e_{y}}\left(t\right)}{r_{v_{x}}\left(t\right)T_{s}}\right), \end{array} \end{array}$$

where *w* is the lane width, L=w during Phase 1, and L=0 during Phase 3.

### 3.4. Parameter Learning

In this section, the parameters defined in Section 3.3 are learned using the experimental data described in Section 2.3. By using the data generated for each set of experimental overtaking maneuvers, an equivalent MATLAB scenario has been created in order to compare the maneuvers done by a real driver and the maneuvers done by the TPC algorithm.

**Figure 10:**
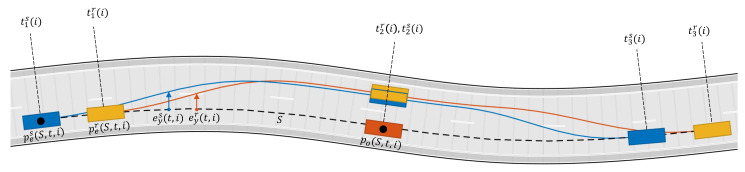
The yellow line/vehicle is the real driver denoted by superscript *r*; the blue line/vehicle is the TPC algorithm simulation denoted by superscript *s*; i is an index for the i-th experimental scenario.

In order to tune the parameters, first we define an objective function which gives us a quantitative measure of how close the real and the TPC algorithm maneuvers are. The objective function is defined as:

(13) $$J\left(k_{1},k_{2},k_{3},k_{4},\delta v,\bar{a},\underline{a}\right)=\sum_{i=1}^{N}\int_{t_{1}^{r}}^{t_{3}^{r}}∥\begin{array}{c} e_{y}^{s}\left(t+\Delta t\left(i\right),i\right)-e_{y}^{r}\left(t,i\right)\\ \delta p^{s}\left(t+\Delta t\left(i\right),i\right)-\delta p^{r}\left(t,i\right) \end{array}∥dt$$

(14) $$\Delta t\left(i\right)=t_{2}^{r}\left(i\right)-t_{2}^{s}\left(i\right),$$

where *N* is the number of experimental scenarios. The purpose of Equation (14) is to synchronize the real and the TPC algorithm maneuvers in time according to a common event, such as the moment that the ego vehicle passes the overtaken vehicle and the two vehicles are aligned wrt to their lateral axis (see Figure 10). The objective function 13 compares the distance of the ego vehicle to the overtaken vehicle and the ego vehicle lateral deviation in the time-frame of the real maneuver to the TPC algorithm maneuver. The parameters have been learned by solving the following optimization problem by means of Monte Carlo simulations:

(15) $${\left(k_{1},k_{2},k_{3},k_{4},\delta v,\bar{a},\underline{a}\right)}^{*}=\arg\min_{k_{1},k_{2},k_{3},k_{4},\delta v,\bar{a},\underline{a}}J\left(k_{1},k_{2},k_{3},k_{4},\delta v,\bar{a},\underline{a}\right),$$

where the star indicates the solution of the optimization problem. The values obtained from this optimization process are shown in Table 1.

## 4. Results and Discussion

To illustrate the efficiency of the proposed method, the TCP algorithm has been tested in simulation on a subset of the experimental scenarios, described in Section 2.3, that was not used for parameter learning in Section 3.4. The experimental scenarios consist of overtaking maneuvers performed by 10 participants. The vehicle speed in these experiments range from 90 km/h to 130 km/h. For each set of data collected in the experiments, an equivalent MATLAB scenario was created, where the ego vehicle is driven by the TPC algorithm instead of the participants. We can observe that this setting is possible only if the participants did not cause the other vehicles to behave differently. Therefore, the scenarios are independent from the trajectory of the ego vehicle. The vehicle model used in the simulations (Figure 7, vehicle dynamics block) has been taken from the MATLAB Vehicle Dynamics Toolbox called the “Vehicle Body 3DOF Dual Track”, with the following parameter values: distance from CoG to the front/rear wheels center $l_{f}=1.58$ m, $l_{r}=1.58$ m, mass m=2100 kg, yaw polar inertia $I_{z}=4000$ kg m${}^{2}$, front/rear cornering stiffness $c_{f}=27\times 10^{3}$ N/rad, $c_{r}=20\times 10^{3}$ N/rad.

To evaluate the performance of the TPC algorithm, different criteria have to be considered. Firstly, the algorithm should be able to successfully accomplish the required task (overtaking maneuver), also providing a certain precision in terms of reference maneuver tracking. Secondly, we have to guarantee a safe maneuver by ensuring an adequate distance from the other vehicles/obstacles in the scenario. Finally, the maneuver should be comfortable, or in other words, the trajectory of the vehicle must be sufficiently smooth.

In terms of task achievement, in all the performed simulations, the TPC algorithm accomplished the overtaking maneuver with satisfactory precision. In terms of safety, the vehicle was able to keep a safe distance from the other vehicles and road boundaries. In this regard, it is worth mentioning that there are two levels of safety measures considered in the algorithm. The first one is in charge of the decision-making block, which decides collision-free maneuvers that the autonomous vehicle should execute (see Section 3.2). The second one is given by the dynamic constraints in the NMPC controller (see Section 3.1). The constraints in the controller guarantee that the ego vehicle trajectory is collision-free as long as it is physically possible to avoid a collision in the considered prediction time interval. Finally, in terms of comfort, the maneuvers performed by the TPC algorithm generally turned out to be smoother than those performed by the human driver. To mathematically assess these three general criteria, the following KPIs were considered:

- KPI1: Root Mean Square (RMS) value of the lateral acceleration
- KPI2: RMS value of the longitudinal jerk
- KPI3: RMS value of the steering velocity (rad/s)
- KPI4: RMS value of lateral deviation from lane center during Phase 2 of the overtake

All the above KPIs can be considered as quantitative measures of the maneuver precision, safety, and passenger comfort. The KPI mean values obtained by simulations on five different scenarios created from the real experiment data are reported in Table 2, where the values given by the proposed TPC MPC algorithm are compared with the ones obtained by the human driver in the real driving maneuvers and with the ones given by a state-of-the-art approach based on the well-known Stanley controller. In this approach, trajectory planning is performed in two steps: (1) a set of points is defined, describing a discontinuous change from the current lane center to the overtaking lane center and back; (2) the set of points is smoothed by means of the Lowess algorithm. Since no scenario variations occurred in the simulations carried out, trajectory planning was performed only at the beginning of the overtaking maneuver. If the road scenario is subject to changes (e.g., arrival of other vehicles or obstacles), this simple trajectory planning approach is no longer adequate and a more sophisticated one, working on-line, has to be used (e.g., A*, RRT). A PID regulator is used for longitudinal dynamics control, and the Stanley algorithm is used for lateral control. Stanley is the controller that won the 2005 DARPA Grand Challenge [12].

**Figure 11:**
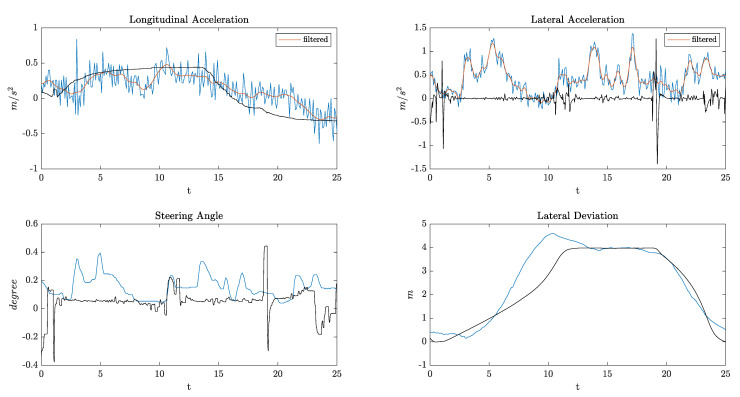
Simulation results. Blue lines: real maneuver data; Black lines: TPC maneuver data.

It can be seen from the data in Table 2 that all the KPIs have lower values in the maneuvers done by the TPC MPC algorithm, which interestingly indicates that the algorithm outperforms the human driver and the TPC Stanley in terms of comfort and precision. Additionally, the Stanley controller has some oscillations at high speeds, since it was originally designed for low-speed maneuvers. As an example, Figure 11 provides longitudinal and lateral accelerations, steering angles, and lateral deviation from the center of the first lane for one of the above-mentioned scenarios. The ego vehicle initial speed was 108 km/h, and the algorithm was able to accelerate and overtake very smoothly with much less lateral acceleration and no overshoot in the lateral deviation compared to the real driver.

Apart from task achievement, safety and comfort, the goal of tuning the algorithm parameters (see Section 3.4) was to imitate the behaviour of a human driver for the overall overtaking maneuver. This similarity can be seen visually in Supplementary Material, where both the real and TCP maneuvers are animated for the same scenario used in Figure 11. Although the experiment scenario in this animation was not used for parameter learning, we can see how similar the two maneuvers are, or in other words, how much the TCP algorithm imitates the human driver.

In summary, these results show that the proposed TPC algorithm and the formulation to tune its parameters using real maneuver data is not only able to perform an overtaking maneuver similar to a human driver, but also explicitly guarantee a safe trajectory in a comfortable manner.

## 5. Conclusions

In this paper, we presented a trajectory planning and control algorithm, based on a Model Predictive Control (MPC) approach, able to work in different road scenarios (such as urban areas and the motorway). The proposed MPC has been designed considering imitation-learning from a specific dataset (real-world overtaking maneuver data), with the aim of getting human-like behavior. This algorithm was used to generate (optimal) trajectories (for lane-change maneuvers and related overtaking). In conclusion, the MPC algorithm is able to imitate the human behavior accurately, also providing better KPI values with respect to the human driver.

As next steps, some possible activities are taken into account. First is the possibility to consider more complex situations and traffic maneuvers, such as double lane-switching or intersections, especially in urban scenarios. For this purpose, a cooperative control approach for AVs can be considered and developed, in order to extend our nonlinear MPC technique to a system with multiple vehicles (AVs and those that are not). Of course, one of the problems to face is the computational feasibility in real time, in which each vehicle computes its own control inputs using estimated states of neighboring vehicles.

## Figures and Tables

**Table 1:** Overtaking maneuver parameters.

$k_{1}$	$k_{2}$	$k_{3}$	$k_{4}$	δv	$\bar{a}$	$\underline{a}$
2	0.5	0.5	1.6	6.5 (m/s)	0.4	−0.3

**Table 2:** KPI mean values ± standard deviation obtained by simulations on five different scenarios created from real experiment data.

Simulation	KPI1	KPI2	KPI3	KPI4
Human driver	0.53±0.20	0.022±0.012	$0.004\pm 1\times 10^{-3}$	0.226±0.041
TPC Stanley	0.46±0.11	0.044±0.015	$0.025\pm 1\times 10^{-2}$	0.47±0.05
TPC MPC	0.21±0.09	$2.3\times 10^{-4}\pm 7.3\times 10^{-5}$	$0.004\pm 9\times 10^{-4}$	0.020±0.005

## Data Availability

Not applicable.

## Supplementary Materials

Animated comparison of the real maneuver and the TPC maneuver is available online at https://doi.org/10.6084/m9.figshare.14828664.v1 (accessed on 9 June 2021).
